# 3D-Printing of Drug-Eluting Implants: An Overview of the Current Developments Described in the Literature

**DOI:** 10.3390/molecules26134066

**Published:** 2021-07-02

**Authors:** Vanessa Domsta, Anne Seidlitz

**Affiliations:** Department of Biopharmacy and Pharmaceutical Technology, Institute of Pharmacy, University of Greifswald, Center of Drug Absorption and Transport, Felix-Hausdorff-Str. 3, 17487 Greifswald, Germany; vanessa.domsta@uni-greifswald.de

**Keywords:** 3D-printing, additive manufacturing, implant, drug-eluting

## Abstract

The usage of 3D-printing for drug-eluting implants combines the advantages of a targeted local drug therapy over longer periods of time at the precise location of the disease with a manufacturing technique that easily allows modifications of the implant shape to comply with the individual needs of each patient. Research until now has been focused on several aspects of this topic such as 3D-printing with different materials or printing techniques to achieve implants with different shapes, mechanical properties or release profiles. This review is intended to provide an overview of the developments currently described in the literature. The topic is very multifaceted and several of the investigated aspects are not related to just one type of application. Consequently, this overview deals with the topic of 3D-printed drug-eluting implants in the application fields of stents and catheters, gynecological devices, devices for bone treatment and surgical screws, antitumoral devices and surgical meshes, as well as other devices with either simple or complex geometry. Overall, the current findings highlight the great potential of the manufacturing of drug-eluting implants via 3D-printing technology for advanced individualized medicine despite remaining challenges such as the regulatory approval of individualized implants.

## 1. Introduction

Many diseases are located in specific regions of the human body. In these cases, a target-oriented treatment with drug-eluting implants is a promising strategy. In this review, the term “drug-eluting implants” is used to describe drug-delivery systems and devices with incorporated drugs that are released in surrounding tissue areas after implantation or insertion.

In contrast to systemic treatment options, a substantial benefit of local administration is the possibility to achieve sufficient drug doses over longer periods of time at sites of action that are difficult to reach, with minimized side effects. Much higher drug doses often have to be administered systemically to achieve therapeutical doses in the targeted tissues depending on the properties of the drug and tissue. Thus, the whole body would be affected by the drug and side effects may occur increasingly. An additional benefit of local drug therapy is the avoidance of frequently repeated dosing, or painful injections, which could increase patient adherence along with the success of therapy [1].

The anatomy of different patients is as diverse as their diseases and the localization of these diseases in the body [2,3,4,5,6]. Consequently, there is a need for individualized therapeutic options for optimal health care which may be given by 3D-printing of personalized drug-eluting implants.

The topic of 3D-printing has received increasing attention in the pharmaceutical and medical sector during the last decades, demonstrated by the timeline result of the medical search platform Pubmed.gov for the search term “3D printing” that had over 3700 results for the year 2020, but only 64 results ten years earlier and only six results in the year 2000. Based on the high degree of freedom related to this manufacturing technique, the production of almost any shape with a high degree of complexity is conceivable. These shapes cannot be achieved by common nonadditive manufacturing methods, and thus 3D-printing is a great opportunity for the growing demand for individualized medicine. These days, multiple 3D-printing techniques and materials are being investigated with regard to manifold characteristics for drug-eluting implants such as shape, surface, microstructure, mechanical properties and drug release behavior.

This review aims to give an overview of the current state of the usage of 3D-printing for drug-eluting implants and highlights the potential of this technique for individualized medicine.

## 2. 3D-Printing Techniques

In 3D-printing, also referred as an additive manufacturing technique, the geometry of three-dimensional objects is generated by the deposition, binding or fusion of materials layer-by-layer. Generally, the printing process starts with the construction of the desired model shape via computer-aided design (CAD). In the design of the objects, geometrical information for every location within the object is given for all three dimensions. For individually adopted designs it is already possible to convert anatomical data from medical imaging, for example by computer tomography (CT) scans or magnetic resonance imaging (MRI), into a three-dimensional model [7,8,9,10,11]. Afterwards, the design is transferred into a readable code for the printer and the actual printing process can be started.

**Figure 1:**
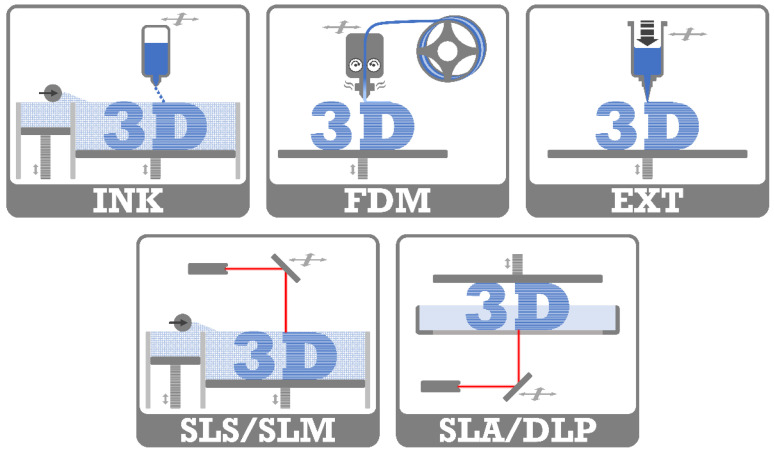
Schematic illustration of the different printing techniques. 3D inkjet printing (INK), fused deposition modeling (FDM), extrusion 3D-printing (EXT), selective laser sintering/selective laser melting (SLS/SLM), stereolithographic 3D-printing/digital light projection (SLA/DLP).

Commonly used 3D-printing techniques in the medical and pharmaceutical field are techniques based on inkjet printing, fused deposition modeling, semisolid extrusion or laser technologies [12,13,14,15,16,17]. They enable the use of different materials such as polymers, ceramics or metals, as well as providing different resolutions for the printed objects [13,15,17,18,19,20]. All of these have advantages and disadvantages as summarized in Table 1 [13,17,20,21,22,23]. A schematic illustration of the printing techniques is shown in Figure 1 and a brief outline of the basic mechanisms is given in the following sections.

### 2.1. 3D Inkjet Printing (INK)

The printing head of an INK system, also referred to as a binder jetting system, is related to traditional inkjet printers. Thermal, electromagnetic or piezoelectric technologies can be used to deposit droplets with high accuracy onto a fine powder bed. The fluids bind the powder layer-wise to form a three-dimensional object. Structures produced by this printing technique typically exhibit high porosity, low mechanical strength and high friability [13]. Regarding the application of implants, the internal structures may be desirable for some indications, but sufficient mechanical stability has to be ensured as well. The printing process itself is relatively fast, but the printed objects need adequate time for drying afterwards [13,24]. Another required postprinting process is the removal of excess powders, which implies the generation of significant waste [13]. Powders or liquid materials used in this 3D-printing technique are already widely used as excipients for dosage forms produced by common manufacturing methods [13], but ink formulations with suitable physical properties, including their density, viscosity and surface tension, still need to be developed to enable good printability [25].

### 2.2. Fused Deposition Modeling (FDM)

In fused deposition modeling, thermoplastic filaments that have previously been prepared by hot-melt extrusion (HME), are fed to a heated nozzle of a movable printhead. The molten materials are extruded and deposited in predefined lines on a build plate. They solidify quickly while the printing process is ongoing for the next layer. Limitations to geometries, such as overhangs, which can be printed self-supported only up to an angle of 45 degrees [26], can be overcome with the use of supporting structures, which are removed after printing. 3D-printers based on FDM technology are widely available and located in a lower price segment compared to other printing techniques [13]. While a variety of filaments are available from different suppliers for standard technical FDM 3D-printing, the desired compositions for medical or pharmaceutical usage need often to be fabricated using a single or twin-screw extruder under convenient conditions for each composition [27]. The high operating temperatures during this printing process, which are mostly over 150 °C, limit the selection of accessible materials due to the risk of degradation of the drug or excipients [13,22]. Especially two-fold thermal stressing during HME and printing process should be considered with this technique. For the manufacturing of implants, biocompatible and biodegradable polymers such as polylactic acid (PLA), poly(lactic-co-glycolic acid) (PLGA) or polycaprolactone (PCL) are commonly used, but many pharmaceutical materials have already been applied to FDM as well, such as cellulose derivates, polyvinyl alcohol (PVA), Eudragit, ethylene vinyl acetate (EVA), polyethylene oxide (PEO) and thermoplastic polyurethane (TPU) [28]. 

### 2.3. Extrusion 3D-Printing (EXT)

Besides FDM, other 3D-printing techniques are based on extrusion mechanisms where the materials are extruded through a nozzle by compressed air, a syringe plunger or a screw, and deposited into the final three-dimensional object layer-by-layer. These semisolid materials can be gels, pastes or molten compositions of the drugs and carriers. The controlled flow of the semisolid materials through the nozzle is challenging and requires suitable properties of viscosity for a successful printing process [13,29]. The types of deposition of the materials are manifold regarding the diversity of available printheads, for example, for high or low temperature usage, for multiple materials or coaxial printing or even heads equipped with UV sources or lasers [29]. Accordingly, the process of solidification is very variable using this printing technique, including processes of crystallization, glass transition, coagulation, drying, precipitation and crosslinking [23]. Compared to other printing techniques, lower resolutions in the range of approximately 0.4–0.8 mm are obtained by extrusion-based 3D-printing depending on the nozzle used [13,20,29]. Printing is mostly processed at low temperatures and is, therefore, suitable for thermolabile substances [13,22,29]. Additionally, high drug loads can be achieved using this technique [15]. 

### 2.4. Laser-Based 3D-Printing

Laser-based 3D-printing technologies utilize the energy of a laser beam or UV light to fuse materials.

The selective laser sintering (SLS) technique uses a laser beam for the selective sintering of a thin layer of finely powdered material into the desired shape, followed by swiping a new layer of powder on the bed. The process is repeated until the complete three-dimensional object is reached and can be removed from the powder bed. The selective laser melting (SLM) technique acts similarly, where the laser melts the powdered materials. The high energy input has to be considered because it could degrade drugs or excipients [13,22]. These high-resolution objects can be printed without the need for supporting structures, due to the surrounding powder [13,22]. However, the remaining unsintered powder requires removal during postprinting processes, and generates waste because reuse is not suitable for unlimited cycles due to possible alteration of the materials [13,23,30]. Metal powders are traditionally used in SLS 3D-printing, but other materials such as polyamide, polystyrene, polycarbonate, polypropylene (PP), PLA, PCL or thermoplastic elastomers can also be used [13,19,31]. 

The polymerization of liquid photopolymers is achieved by the exposure of a focused UV laser or the use of digital light projection (DLP) in stereolithographic 3D-printing (SLA). In this way, the designed object is created layer-by-layer on a movable platform. This printing technique has a high level of accuracy and resolution related to the spot size of the laser [13,22,23]. For SLA printed objects, postprocessing by further curing of the object is usually necessary [13,17,31,32]. This technology is limited by the need for photopolymers, which are relatively uncommon in pharmaceutical manufacturing, and the potential for residual resins that may imply risks of toxicity [13,17,19,33].

## 3. Drug Loading Mechanisms

These days, many different mechanisms have been implemented for the loading of drugs into 3D-printed implants. All methods aim to achieve a reproducible loading of intact drugs into the implant in effective therapeutical doses. The loading mechanisms can be divided roughly into mechanisms where the drug is incorporated into the printing matrix before or during the printing process, and those in which the implants are 3D-printed first and drug loading is performed afterwards.

Typical for FDM 3D-printing, the drugs can be incorporated in the filaments while these are prepared by HME. For this purpose, the drugs are previously blended with the polymer composition or coated onto polymer pellets by using an oil casting method [31,34,35,36,37,38,39,40,41,42,43,44]. The drug should be homogenously dispersed in the filament for controllable drug loading of the implants because no mixing effects occur during the printing process. Filaments with a less homogenous distribution of the drug were produced by Holländer et al. [45], where a part of the pure polymer was melted in the extruder first and the drug and remaining polymer were added subsequently into the melt. Depending on the used extruder equipment, more or less extensive mixing occurs during the extrusion process when using a single- or twin-screw extruder [27]. This highlights the importance of suitable feeding materials for the quality of filaments, and thus for the printed implants. Similar requirements of homogeneity have to be met by the blended starting materials for other extrusion-based 3D-printing techniques regardless of whether heat is supplied or not [36,46,47,48,49,50]. Besides HME, drugs can also be incorporated into the filament matrix by immersing drug-free filaments in highly concentrated drug solutions. The used solvents would need to dissolve sufficient amounts of the drug to enable the incorporation and at the same time not dissolve the polymer material to maintain the filament structure. Achievable drug loadings are limited in this diffusion-based technique by the properties of the drug, solvent and polymer. For example, Qamar et al. [51] loaded PVA filaments to a drug amount of 5 ± 1% by immersion in an ethanolic solution of ciprofloxacin hydrochloride, but only reached 3 ± 1% drug loading for PP filaments by the use of an aqueous solution of the same drug. Furthermore, the content uniformity was not satisfactory for those filaments [51]. An advantage of this immersion procedure is that the drug is not exposed to high temperatures as with HME. However, this method should probably play a minor role in future developments considering that heat exposure affects the drug during the FDM 3D-printing process anyway, and that the highly concentrated drug solutions are costly, waste generation occurs and there is a limit to achievable drug loading.

For the incorporation of drugs into the printed matrix during DLP, the drugs have to be dissolved or suspended in the liquid photopolymers. However, the impact on the cross-linking behavior should be examined [52,53].

3D inkjet printing enables the incorporation of drugs into the implant during the printing process as they can be added to the powder bed or binder solution [54,55,56,57,58,59]. The drugs used in this method have to be stable and soluble within suitable concentrations in the solvents of binder solutions, which often contain acetone, methanol or ethanol [54,55,56,58]. Moreover, printers with multiple ink cartridges can incorporate one or multiple drug solutions into the implant independent of the binder solution [57,60]. All of these possibilities demonstrate the progression of 3D-printing over time in comparison to manual drug positioning, by pipetting, that was used in the initial studies of Wu et al. [61] presented in 1996.

As a postprinting procedure, finished drug-free 3D-printed implants can be loaded with drugs by a coating procedure [62,63]. Furthermore, drug solutions can be applied dropwise on the printed object [64,65,66] or the whole implant can be immersed in a drug solution, often under vacuum, to absorb the drug [67,68,69,70,71,72]. These methods are often very time-consuming and only porous structures [67,68] enable the possibility of suitable drug concentrations in the inner parts of the implant. Other, more exceptional, postprinting drug loading mechanisms are the incubation of implants with sublimated iodine [73], drug loading with supercritical carbon dioxide [74] or the manually filling of powdered drug or drug-loaded alginate gel into previously 3D-printed hollow or reservoir structures [75,76,77,78].

All in all, the drugs that are incorporated into the implant matrix before or during the printing process have to withstand all conditions of the preparations and the actual printing process. Consequently, some sensitive drugs are excluded from those mechanisms due to their degradation properties. For example, thermo-labile drugs have to be excluded for most HME and FDM 3D-printing processes involving polymers with a high melting temperature.

Due to the incorporation of drugs in the starting materials, the homogenous distribution of the drug is more easily achieved and less dependent on the loading parameters such as time or temperature during an immersion process. Additionally, long-term releases of the drugs are conceivable due to long diffusion distances from the center of the matrix to the boundary to the surrounding tissue or body fluid. For this purpose, incorporation of the drug throughout the entire implant should be ensured, which is not easily achievable by postprinting loading procedures with fairly large objects. However, besides the missing exposure of sensitive drugs to printing conditions, an important advantage of the postprinting loading mechanisms is the possibility of performing postprocessing steps, for example washing steps, without influencing the drug.

In summary, different drug loading mechanisms are applicable, but possess different limitations for some applications and should be chosen depending on the drug properties, printing material, 3D-printing technique and desired properties for the final product, including drug release characteristics of the implant.

## 4. Current Medical and Pharmaceutical Applications of 3D-Printing

The 3D-printing of drug-eluting implants represents just a very small part of the possible applications of this manufacturing technique in the medical and pharmaceutical fields.

A popular topic is the usage of 3D-bioprinting for tissue engineering and organ printing [79,80,81,82], but also anatomical models have a high level of interest. They enable easier education of patients [83,84,85] as well as a multidimensional perspective for planning and training [86,87,88,89,90,91] of surgery for physicians. 3D-printed replicates of patients’ body parts can possibly improve the procedure during surgery as an intraoperative guidance tool [92,93]. Furthermore, on-demand 3D-printing of customized orthosis, prostheses or implants can fulfill the requirement of exactly fitting the patients´ conditions [94,95,96,97,98,99]. Drug-loaded 3D-printed products have been developed in various fields such as oral medication, for example, tablets or capsules with immediate or sustained release properties of one or multiple drugs in just one device [100,101,102,103,104,105], oral films [106,107], transdermal microneedle systems [108,109,110] or implants, which are highlighted in this review.

In addition to the directly 3D-printed medical or pharmaceutical products, this technology can also be used for the 3D-printing of individual molds [111,112,113,114]. Thus, beneficial freedom for the desired shape can be combined with materials that are unsuitable for direct 3D-printing at present or can be further implemented in manufacturing procedures or development steps.

## 5. 3D-Printing of Drug-Eluting Implants

**Figure 2:**
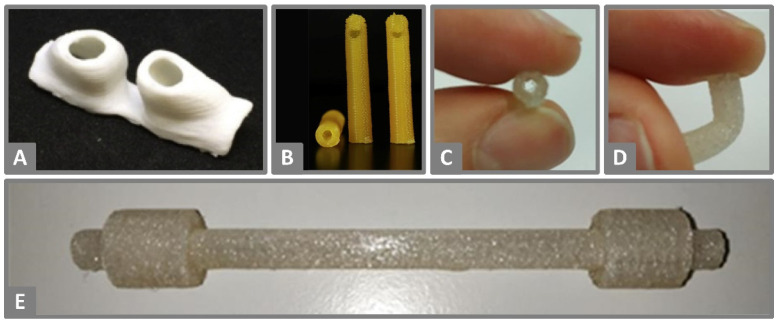
(**A**) FDM 3D-printed personalized nasal support of PLA for postoperative cleft rhinoplasty. Reprinted from Boyer et al. [62], Copyright (2018), with permission from Elsevier. (**B**) FDM 3D-printed 14-F catheter tips with a length of 5 cm of PLA loaded with methotrexate. Reprinted from Weisman et al. [119], Copyright (2019), with permission from Elsevier (**C**,**D**) FDM 3D-printed catheters made of TPU demonstrating their flexible properties. (**E**) FDM 3D-printed catheter with integrated cuffs made of TPU (**C**–**E**). Reprinted with permission from Mathew et al. [41], Copyright (2019), American Chemical Society.

**Figure 3:**
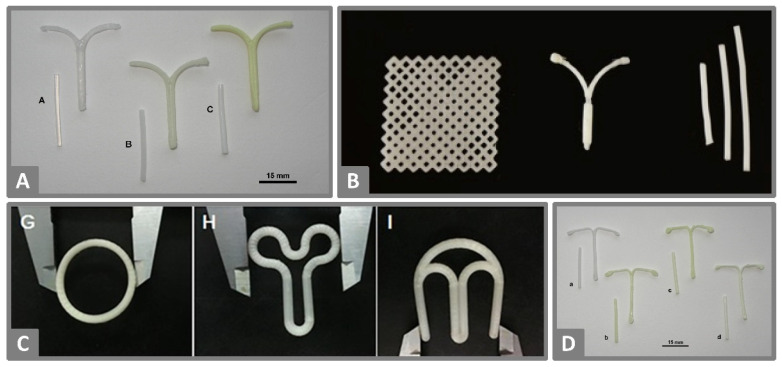
(**A**) EVA filaments prepared by HME loaded with 0, 5 and 15% indomethacin (left to right) and the corresponding FDM 3D-printed prototypes of an IUD. Reprinted from Genina et al. [34], Copyright (2016), with permission from Elsevier. (**B**) FDM 3D-printed PCL meshes loaded with estrogen (left), IUD loaded with progesterone (middle) and subdermal implants (right). Reprinted from Tappa et al. [42], licensed under CC BY 4.0. (**C**) FDM 3D-printed O, Y and M-shaped vaginal rings based on a composition of PLA, PCL and PEG loaded with progesterone. Reprinted from Fu et al. [39], Copyright (2018), with permission of Elsevier. (**D**) PCL filaments prepared by HME loaded with 0, 5, 15 and 20% indomethacin (left to right) and the corresponding FDM 3D-printed prototypes of IUD. Reprinted from Holländer et al. [45], Copyright (2016), with permission from Elsevier.

**Figure 4:**
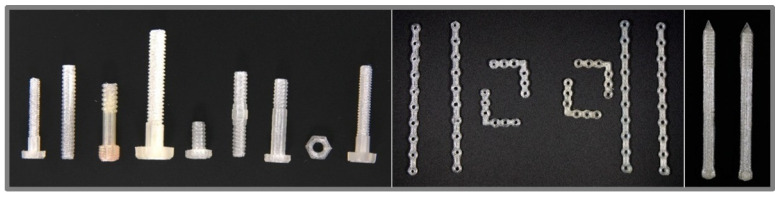
FDM 3D-printed orthopedic screws, plates and pins of PLA. Reprinted from Tappa et al. [35], licensed under CC BY 4.0.

**Figure 5:**
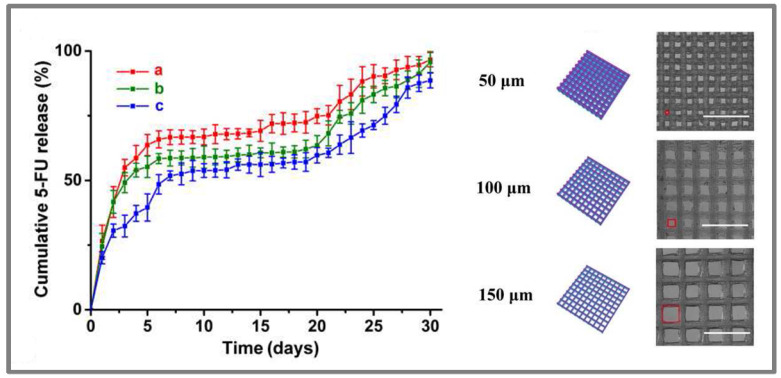
Release profiles of 5-fluorouracil, simulated diagram and microscopic image of scaffolds with varying aperture sizes (a—150 µm, b—100 µm, c—50 µm), that were 3D-printed via an E-jet system. Samples were incubated in 3 mL PBS pH 7.4 at 37 °C with shaking at 100 rpm. Reprinted from Yang et al. [48], Copyright (2020), with permission from Elsevier.

**Figure 6:**
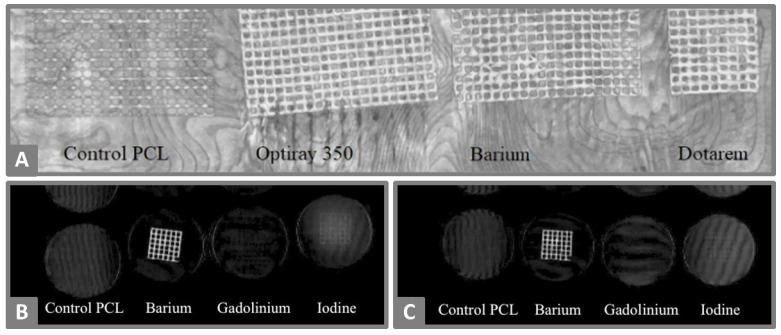
(**A**) Maximum intensity project coronal reconstruction of 3D-printed meshes of PCL loaded with barium, gadolinium or iodine as a contrast agent, and control a drug-free mesh. (**B**,**C**) Coronal volume rendering of meshes with and without contrast agent incubated in 37 °C agar solution for one day (**B**) or seven days (**C**). Reprinted from Ballard et al. [140], licensed under CC BY 4.0.

**Figure 7:**
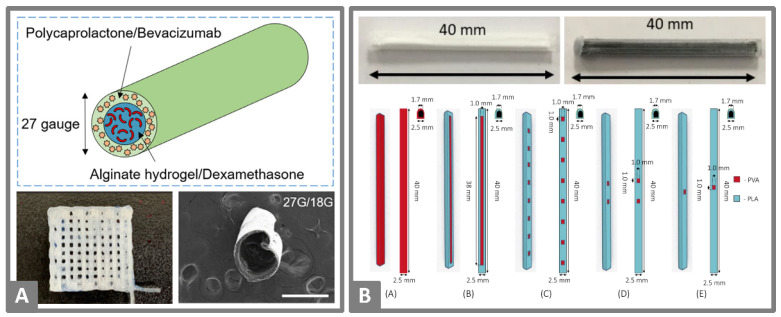
(**A**) Schematic structure of a coaxial extruded rod of PCL with bevacizumab in the outer shell and afterwards injected with alginate gel loaded with dexamethasone in the center (top). PCL shell tube loaded with bevacizumab (left) and 3D-printed meshes of those shell tubes (right, scale: 200 µm). Reprinted from Won et al. [77], Copyright (2020), with permission from Elsevier. (**B**) FDM 3D-printed subcutaneous implants manually filled with methylene blue (left) or ibuprofen sodium (right), and schematic images of different implant designs for 3D-printing implant shells with PVA (red) and PLA (blue). Reprinted from Stewart et al. [75], licensed under CC BY 4.0.

In the following sections examples of 3D-printed drug-eluting implants described in the current literature are presented. For a better overview, the implants were grouped into stents and catheters, gynecological devices, devices for bone treatment and surgical screws, antitumor devices and other devices with either simple or complex geometry. Additionally, the main facts regarding those implants are briefly summarized in Table 2, Table 3, Table 4, Table 5, Table 6, Table 7 and Table 8 and selected images are given in Figure 2, Figure 3, Figure 4, Figure 5, Figure 6 and Figure 7.

### 5.1. Stents and Catheters

Implanted stents should ensure the opening of vessels or cavities, and catheters should enable drainage or the application of fluids. With the insertion of a foreign object, the risk of infection is high [115,116,117]. In research on 3D-printed stents and catheters, attempts to manage this risk include use of antimicrobial drugs such as nitrofurantoin, gentamicin, penicillin or tetracycline [31,38,40,41,62,118,119]. Additional to the inhibition of microbial growth, tube-shaped implants have to fulfill high standards for their mechanical stability over a longer period of time [120,121].

The proof-of-concept study of Sandler et al. [31] demonstrated the successful incorporation of the antimicrobial drug nitrofurantoin (5% *w/w*) into a 3D-printing process by HME of PLA filaments. A test on a biofilm formation of *Staphylococcus aureus* (*S. aureus*) showed an inhibitory effect of the 3D-printed discs containing nitrofurantoin. Moreover, these devices had a stronger inhibiting effect on bacterial growth than placebo discs with a separate drug-solution on top. This highlights the potential of 3D-printing for the fabrication of implantable medical devices with a lower risk for infections. Similar studies by Weisman et al. [38] confirmed these findings. 3D-printed objects loaded with gentamicin sulfate (1–2.5% *w*/*w*) or methotrexate (2.5% *w*/*w*) demonstrated growth inhibition of *Escherichia coli* (*E. coli*) and osteosarcoma cell decrease, respectively. The printing process did not seem to reduce antibiotic effectiveness because drug-loaded filaments and printed parts showed comparable bacterial growth inhibition. The authors of this study highlighted the application of 3D-printing for the manufacture of several objects, such as discs, beads and catheters that were actually printed in these studies. Moreover, the authors claim that any medical construct could be printed, even with complex geometries. Additionally, the drug loading method by HME can be transferred to other drugs. Consequently, the free combination of drugs and shapes permits customized and tailored treatments in many medical fields.

A high level of complexity regarding the shape of the 3D-printed objects was reached by Boyer et al. [73] by printing vascular Y-stents with an internal mesh structure. These stents and simple meshes were printed with water-soluble PLA filaments, and postprocessed by optional cross-linking and additional iodization. The iodine was supposed to combine with an antimicrobial effect and high visibility in CT imaging. Both effects were shown in this study, and the iodization provided a method for the determination of the exact localization of the 3D-printed implant.

Currently, many stents for support in lip and palate surgery use the concept of one-size-fits-all but Mills et al. and Boyer et al. worked on solutions for higher personalization using the FDM 3D-printing technique [40,62]. Boyer et al. [62] used photogrammetry to achieve the ideal fit for patients’ contours (Figure 2A). A series of photos was taken of a negative formed by injection molding of a nostril and merged into a 3D computer model. During postprocessing, structural modifications were feasible before the printing process with PLA. The 3D-printed stents were coated with the water-soluble polymer PVA containing penicillin to a drug load of 1.3% (*w*/*w*). These successfully inhibited the growth of *E. coli*, similar to the stents of Mills et al. [40], where the drug gentamicin sulfate (1–2.5% *w*/*w*) was incorporated in the self-manufactured PLA filaments by HME before printing.

Weisman et al. [119] created catheters by FDM 3D-printing with antibiotic and chemotherapeutic-loaded PLA filaments (Figure 2B). These catheters showed an initial burst release of the drugs followed by a steady release into simulated body fluid until the end of the test after five days. This study demonstrated the potential of 3D-printing for hollow implants using catheters. Under in vivo conditions, the application of PLA-based catheters to patients is difficult due to the rigid nature of this polymer [41]. To overcome this, Mathew et al. [41] developed different catheter designs that were 3D-printed with the flexible polymer TPU (Figure 2C–E) containing the antibiotic drug tetracycline hydrochloride. The incorporated drug concentrations of 0.25%, 0.5% and 1% (*w*/*w*) did not seem to influence the elastic modulus of the filaments. While all three drug loads resulted in an inhibition effect on the growth of *S. aureus*, the effect was more pronounced when the highest drug load of 1% (*w*/*w*) tetracycline hydrochloride was used. For this drug load, the growth inhibition was still present after 10 days of release in 1 mL phosphate-buffered solution (PBS). Even longer antibacterial effectiveness is likely since only 4% of the total drug load was released at this time.

Considering the presented research for 3D-printed stents and catheters, the focus seems to be on the incorporation of antimicrobial drugs to avoid side effects associated with infections after implantation. Antimicrobial efficacy was proven for the 3D-printed products, but in some cases the printing of very simple shapes, or the use of rigid materials, indicates the proof-of concept characteristic of those studies. Furthermore, individual studies demonstrated the opportunities of 3D-printing flexible catheters, the possible verification of implant placement using CT-imaging, as well as personalization in the form of individualized shapes or different drug loads. Consequently, the remaining challenge for the future will be to combine the achieved findings in a 3D-printed stent or catheter that fulfils the requirements of a safe and easy application and provides the benefits of personalization.

The main facts from selected literature regarding 3D-printed drug-eluting stents and catheters, including materials used, methods and objectives, are summarized in Table 2.

**Table 2:** List of selected 3D-printed drug-eluting stents and catheters described in the literature.

Implant Shape	Material	Drug	Drug Loading	Printer Type	Objective	Source
disc	PLA	nitrofurantoin	HME	FDM	incorporation of an antimicrobial drug in 3D model structure to inhibit biofilms	Sandler et al., (2014) [31]
disc, bead, catheter	PLA	gentamicin sulfate, methotrexate	HME	FDM	3D-printing of different constructs with antibiotic or chemotherapeutic-eluting filament	Weisman et al., (2015) [38]
nasal stent	PLA	gentamicin sulfate	HME	FDM	personalized nasal stents with bioactive properties in cleft surgery	Mills et al., (2017) [40]
nasal stent	PLA, PVP	penicillin	postprint: dip coating	FDM	postoperative patient-specific nasal supports with bioactive agents	Boyer et al., (2018) [62]
mesh, Y-stent	PVA	iodine	postprint: gaseous incubation	FDM	antimicrobial and highly visible (CT image) meshes/stents of iodized (cross-linked) PVA	Boyer et al., (2018) [73]
catheter	PLA	gentamicin sulfate, methotrexate	HME	FDM	3D-printing of bioactive laden bioabsorbable catheters	Weisman et al., (2019) [119]
catheter	TPU	tetracycline hydrochloride	HME	FDM	incorporation of an anti-infective drug into 3D-printed catheters	Mathew et al., (2019) [41]

### 5.2. Gynecological Devices

Intrauterine devices (IUD) are inserted into the woman’s uterus for effective and reversible long-term contraception. Even when the use is considered safe in general, complications such as pelvic inflammatory disease or irregular bleeding can occur [122]. Moreover, a displacement of the device is found in up to 25% of IUD-wearing women, and uterine perforation is a rare but critical complication [123].

Goldstruck et al. [124] described high variability in size and shape of the endometrial cavity. This points out how difficult it is for an IUD to match such highly variable dimensions. The devices need to fit well in the endometrial cavities and should adapt to continuous changes of shape due to uterine muscular activity. 3D-printing could be a suitable method to improve these criteria due to its simple shape adjustment.

The implementation of new materials to a 3D-printing technique holds many challenges, demonstrated by research on the printability of twelve different EVA grades for 3D-printing of a T-shaped IUD (Figure 3A) and subcutaneous rods by Genina et al. [34]. Many adjustments were required for the HME and FDM 3D-printing process to find suitable parameters for each grade that differed in the content of vinyl acetate and thus in their polarity, adhesion, crystallinity, flexibility and melting point. For example, some compositions were limited for printability by FDM due to an unsuitable melt index or the high elasticity of some filaments. This excluded them from successful feeding to the print head, and the mechanical properties were inadequate for continuous extrusion of the molten material through the nozzle. A too low melt index, which increases with an increased vinyl acetate content, resulted in insufficient filament pressure for the extrusion of the molten mass. Only drops were extruded instead of a continuous strand when the melting index was too high. In the end, five EVA grades were printable, and the most promising one was loaded with 5 or 15% of indomethacin as a model drug, 3D-printed into prototypes and used for further investigations. HME was performed at a temperature of 110 °C, which is lower than the melting point of indomethacin at 160 °C, but the 3D-printing was performed at a higher temperature of 165 °C. Consequently, the drug had a crystalline state in the filaments and an amorphous or dissolved state in the implants. This was investigated by differential scanning calorimetry (DSC) and x-ray diffraction (XRD), and also indicated by the yellow coloration of the products after the printing process. This highlights the importance of the drug state in the polymers, which could explain faster drug release of printed prototypes examined in release studies over 30 days than the release of the drug from filaments with the same dimensions.

Further studies by Holländer et al. [45] demonstrated the relevance of the solid-state of the incorporated drug. HME and FDM 3D-printing of PCL with 5, 15 or 30% indomethacin at 100 °C resulted in a yellowish T-shaped IUD (Figure 3D) due to the fact that indomethacin was partly dissolved in the polymer. The drug release behavior in 200 mL 0.9% saline solution at 37 °C over 30 days started with an initial burst release followed by sustained release and was affected by the drug load and solid-state. The fastest release was observed for the composition with the highest ratio of amorphous drug, which was found in printed products with a 5% drug load, whereas the highest drug load of 30% showed slower drug release due to the higher crystalline drug amount. The drug release from PCL occurs via diffusion of the drug through the polymer as well as the polymer degradation process itself. The determination of the degradation properties resulted in low degradation rates of ≤3.12% over the 30 days of the release test. Therefore, diffusion seemed to be the dominant release mechanism accompanied by a less pronounced influence of PCL degradation. The high drug load of 30% indomethacin did not only influence the drug release but also altered the quality of received product surfaces, where small cracks were visible. The observed solid-state of indomethacin did not seem to be stable during storage, and recrystallization occurred in printed samples. Additionally, the researchers reported trouble with the low adhesion properties of PCL to the build plate, which they solved by using a polyimide tape as a base and printing a drug-free raft that needed to be removed after printing. The authors stated that polymer adhesion to the build plate is influenced by many parameters such as the complex geometry of the printed object, the build plate surface, the printing temperature and the environmental temperature, which could be improved by a printer enclosure.

Whereas Genina et al. [34] and Holländer et al. [45] benefited from the color indicator for amorphous or dissolved indomethacin during their 3D-printing research of IUD, Tappa et al. [42] first loaded PCL filaments with relevant hormones for gynecological therapy by HME. The relatively low melting temperature of biocompatible PCL enabled 3D-printing of discs, surgical meshes, subdermal rods, IUDs and pessaries with 1% (*w*/*w*) estrogen or progesterone (Figure 3B), which showed an extended-release at least over the investigation period of one week.

The 3D-printed O, Y and M-shaped vaginal rings (Figure 3C) developed by Fu et al. [39] had complex compositions to functionalize the implant properties by the choice of ingredients. The final product contained 5% (*w*/*w*) progesterone, PEG 4000, Tween 80, PLA and PCL. The final preferred ratio of the two biodegradable polymers PLA and PCL (8:2) was chosen based on the mechanical properties. Higher amounts of PLA resulted in stiff and easily breakable filaments when loaded with progesterone, whereas higher amounts of PCL increased elasticity. The amorphous state of the hydrophobic drug in the 3D-printed vaginal rings was expected to result in better release properties than the crystalline drug. Additionally, drug release rate should be increased by better wettability of the product by the use of the surfactant Tween 80 and the pore-forming agent PEG 4000, which was fused into a solid dispersion with progesterone before blending with the other ingredients for HME. Tween 80 and PEG 4000 in combination accelerated drug release compared to tested vaginal rings without each excipient. Other soluble additives such as sodium chloride, sucrose or citric acid were not able to achieve a comparable effect. The different area-volume ratios of the printed shapes affected release properties and showed the fastest drug release for O-shaped rings. From the presented study it can be expected that the final 3D-printed products are easily insertable because of their ability to withstand compression without breaking and reversion to the original shape, and that progesterone release is sustained over a minimum of seven days. The authors pointed out that not only the shape was easily adjustable for prospective personalization, but custom drug doses were feasible with this low-cost procedure.

PP is a typical material used for the production of surgical vaginal meshes in the treatment of pelvic organ prolapse or stress urinary incontinence. It is a relatively rigid material and hence does not meet the requirements for this application site due to high motility. Domínguez-Robles et al. [43] 3D-printed bacteriostatic vaginal meshes of flexible TPU with different loads (0.25–1%) of the antibacterial drug levofloxacin for the treatment of pelvic organ prolapse or stress urinary incontinence. Mechanical testing proved the elastic properties of the meshes in comparison to 3D-printed meshes of PP, which showed early appearance of fractures due to stretching. TPU meshes could be elongated more than three times their original length with only minor fractures without completely breaking and seemed to be a suitable material for applications with high motility requirements. Farmer et al. [125] also demonstrated the flexible mechanical properties of FDM 3D-printed TPU meshes loaded with 0.25% or 1% estradiol and studied the effects of the printed mesh geometries on the mechanical properties.

Similar requirements on implant elasticity were met by Zhao et al. [67] with a special 3D-printing technique for cone-shaped cervical tissue implants. During low-temperature deposition manufacturing (LDM), a polyurethane solution was extruded at a low environmental temperature of −30 to −40 °C for rapid solidification. By varying the wire space, different sizes of macropores could be achieved. Subsequent freeze-drying of printed constructs led to the formation of additional micropores. The implant surface was treated with oxygen plasma to modify hydrophilicity before drug loading by immersion in a protein solution under negative pressure. The macro and microporous structures enabled tissue-mimicking of mechanical properties and enlarged the surface, which was loaded with an anti-HPV-protein to prevent acute virus infection after cervical conization.

The study objects are beneficial since most of them comply with requirements for gynecological products regarding their mechanical properties, which are mainly influenced by the choice of appropriate starting materials. Whereas some studies used only model drug substances, in several cases relevant drugs, for example hormones, were incorporated with different drug loads into the devices. The implants were mostly printed in relevant shapes for the actual application site, and personalization was attempted by printing different shapes. However, implementation of personalized shapes adapted to real anatomical data of the patients, and the possibility to check the right placement of the devices initially and during regular use, would be beneficial in the future. 

The main facts from selected literature regarding 3D-printed drug-eluting gynecological devices, including materials used, methods and objectives, are summarized in Table 3.

**Table 3:** List of selected 3D-printed drug-eluting gynecological devices described in the literature.

Implant Shape	Material	Drug	Drug Loading	Printer Type	Objective	Source
t-shaped IUD, s.c. rod	EVA	indomethacin	HME	FDM	3D-printed implants of different grades of EVA	Genina et al., (2016) [34]
t-shaped IUS	PCL	indomethacin	HME	FDM	long-lasting biodegradable implants with different drug loads and sustained drug release	Holländer et al., (2016) [45]
mesh, s.c. rod, IUD, pessary	PCL	estrogen, progesterone	HME	FDM	hormone-eluting customizable and biodegradable 3D-printed implants	Tappa et al., (2017) [42]
O/Y/M-shaped vaginal ring	PLA, PCL, PEG	progesterone	prefused HME	FDM	3D-printed vaginal rings in various shapes for personalization and controlled drug release	Fu et al., (2018) [39]
vaginal mesh	TPU	levofloxacin	HME	FDM	antibacterial vaginal meshes with suitable mechanical properties	Domínguez- Robles et al., (2020) [43]
cervical tissue implant	PU	anti-HPV- protein	postprint: immersion	LDM	3D-printing with LDM and freeze-drying for porous and elastic tissue implants	Zhao et al., (2020) [67]
mesh	TPU	estradiol	HME	FDM	influence of mesh geometry on the mechanical properties of 3D-printed surgical meshes	Farmer et al., (2021) [125]

### 5.3. Devices for Bone Treatment and Surgical Screws

In the treatment of chronic osteomyelitis, surgical interventions including debridement of infected dead tissue, are necessary as well as antibiotic therapy [126]. Antibiotics are applied over weeks in intravenous therapy in such cases, but still infection relapse poses a problem in these patients. Such relapse might be overcome with higher local drug concentrations in affected tissues and lower systemic drug levels by local treatments [126]. Furthermore, implant-associated infections can be caused by contamination during surgical procedures [127]. Antimicrobial drugs, for example isoniazide, rifampicin, minocycline, gentamicin or vancomycin have been used for the 3D-printing of implants in the sector of bone treatment in several studies [35,44,46,54,55,56,57,59,60,68,72,128,129,130,131,132], and glucocorticoids such as dexamethasone or prednisolone have been incorporated in those products as well [63,133,134,135]. 3D-printing could be used for the manufacture of individually adjusted implant shapes to treat or prevent infection of the bone and surrounding tissues.

In 2009 Wu et al. [55] used an inkjet 3D-printing technology for the development of programmed multiphasic drug release from multidrug implants for the treatment of bone tuberculosis. In previous studies, the researchers demonstrated that 3D-printed implants of defined complex multilayered structures offer opportunities for more controllable drug release [54,128]. In their enhanced implant design, the PLA-based implant was a concentric cylinder with four circular layers alternately loaded with the antituberculosis drugs isoniazid and rifampicin. Release studies reported by the authors illustrated the predicted orderly release of the drugs from the outer to the inner layers with a pulsatile behavior in vitro and in vivo. The implants were inserted in the femoral bones of rabbits and no local or systemic infection was monitored. Moreover, drug levels were found in samples of the bone in the predicted manner of the programmed design, but only at low levels in the arterial blood, which is a promising finding with regard to low systemic exposure. In subsequent studies, Wu et al. [56] developed similar 3D-printed multilayer implants with the drugs levofloxacin and tobramycin for the treatment of chronic osteomyelitis and tested those in vitro and in vivo in an animal model. Furthermore, studies of Li et al. [130] and Zhu et al. [129] in rabbits confirmed the findings of sufficient drug levels in bone surrounding tissues over 12 weeks combined with low blood concentrations for 3D-printed scaffolds loaded with the antibacterial drugs isoniazid and rifampicin.

Farto-Vaamonde et al. [135] developed another strategy for the controlled drug release of dexamethasone and prednisolone loaded 3D-printed scaffolds for bone repair. They demonstrated the large influence of the timing of drug loading on the release profile of FDM 3D-printed products. The drug loading of the filaments by an immersion method before printing resulted in sustained drug release, explainable by the encapsulation of the drug in the polymer during melting. In contrast, rapid drug release properties were reached by the immersion of the final 3D-printed product in the drug solution. Upon combination of the two methods, the drug concentration primarily loaded in the filaments was altered by the second immersion step of the printed object, but the combination of both methods offered differential release properties within one device.

Bioceramics are promising materials for the reconstruction of bone defects and combined with 3D-printing and medical imaging they seem to be a promising approach in the treatment of irregular bone defects. Whereas Gbureck et al. [68] studied the drug adsorption and desorption properties of 3D-printed ceramics, which were drug-loaded by immersion of the printed products in different concentrated antibiotic solutions after the printing process, Vorndran et al. [60] developed a multijet printing technique at low temperatures for bioceramic implants loaded with the model drugs vancomycin, heparin or recombinant bone morphogenic protein 2 (rhBMP-2). In this inkjet printing technique, fluid from different cartridges containing either binder solution or drug solutions was printed in a predetermined process. The drug was deposited accurately in predefined locations independent of the basal shape of the implant. The strategies for release modification in this study were the different distribution of the drug in a homogenous, central depot or graduated manner, as well as using chitosan in the binding solution or mixing the ceramic powder with HPMC. The effect of these modifications highlighted the large dependence of drug localization on release kinetics. An advantage of the used printing technique is the relatively low temperature that the drugs are exposed to, but studies of Inzana et al. [136] recognized that the concentrations of phosphoric acid, which were used in the binder solution, pose the risk of residual acidity and consequent cytotoxicity or drug degradation. The authors optimized the binder solution to a phosphoric acid concentration of 8.75 %, added 0.25 % Tween 80 and tested the rifampicin and vancomycin containing scaffolds of this composition successfully in a mouse model [57].

Another technique that did not require high temperatures for ceramic or glass scaffolds in a second sintering step, was presented by Wu et al. [133]. The paste extrusion 3D-printing of mesoporous bioactive glass (MBG) with PVA as a binder offered a low-temperature procedure for 3D-printing of scaffolds with highly ordered mesoporous channel structures, good mechanical strength and sustained drug delivery properties for bone regeneration.

Thus, the choice of the appropriate printing technique and material enables the use of various drugs including thermo-labile drugs. This was also demonstrated by Lee et al. [137], where PCL discs loaded with rifampicin were 3D-printed via an extrusion technique at a low temperature of 60 °C to maintain antibacterial activity of the heat-sensitive drug. The use of more heat resistant drugs can offer more opportunities in the choice of polymer, including highly processable ones, without a loss of efficacy. For example, Weisman et al. [44] demonstrated bacterial growth inhibition of *E. coli* for osteomyelitis treatment with gentamicin-loaded PLA constructs doped with halloysite nanotubes after thermal manufacturing by HME at 170 °C and FDM 3D-printing at 215 °C.

Besides the already mentioned materials, metals can also be used for 3D-printing of implants in bone treatment, manufactured via SLM possibly followed by a coating process to include the drug. Han et al. [132] coated 3D-printed scaffolds of cobalt-chromium-molybdenum (CoCrMo) successfully with the antibacterial drug gentamicin by an electrophoretic deposition method, whereas Poudel et al. [63] used an air-brush technique for drug coating of previously 3D-printed objects made of stainless steel. Both enabled the combination of the mechanical properties of metal materials with drug release for improved therapy and an easily adjustable shape by 3D-printing.

Mechanical properties are very important for surgery fixation implants and were therefore of major interest in research on printed drug-loaded fixation constructs produced by FDM 3D-printing with PLA or solution-extrusion based 3D-printing with PCL and nano-hydroxyapatite (nHA) [35,46]. Tappa et al. [35] printed a wide variety of fixation implants like different screws, pins and plates (Figure 4) as well as more simple constructs for mechanical testing using gentamicin and methotrexate-loaded PLA filaments. Besides the antibacterial and chemotherapeutic effect, the authors were able to demonstrate the dependence and adaptability of mechanical properties on the printing and design parameters. The flexural strength was mostly independent of the infill ratio but altered by the orientation in the X, Y or Z-axis due to the alignment of the layer structure. In contrast, the compression strength was strongly influenced by the infill rates but not by the orientation. The incorporation of drugs decreased both flexural and compression strength. By the addition of the drug, the compression strength was reduced by 48% or 42% for gentamicin or methotrexate containing samples, respectively, compared to the drug-free controls. The modifiable mechanical properties of 3D-printed fixations could possibly match the needs for different types of bones. Moreover, the use of degradable biomaterials might make the removal of the implants during revision surgery unnecessary.

The successfully incorporated drugs in the presented studies are relevant for the desired application into bone due to their antimicrobial or anti-infective properties. Several studies enabled the 3D-printing process at lower temperatures in order to assure processability of thermolabile drugs. The adaption of the implant shape to the physiological condition, for example, the exact shape of the bone defect, is still pending. However, the realization of these shapes should be feasible using the applied 3D-printing technology. The performed in vitro tests, including in vitro release studies, enabled first estimations on the properties and performance of the 3D-printed implants, but due to the highly standardized conditions of these tests, the results cannot be transferred directly to their estimated performance in human patients. Studies with animal models, which have been performed in some cases, such as the implantation of the 3D-printed objects in rabbits, present a necessary step towards the necessary trials in humans.

The main facts from selected literature regarding 3D-printed drug-eluting implants for bone treatment and surgical screws, including materials used, methods and objectives, are summarized in Table 4.

**Table 4:** List of selected 3D-printed drug-eluting implants for bone treatment and surgical screws described in the literature.

Implant Shape	Material	Drug	Drug Loading	Printer Type	Objective	Source
cylinder	ceramics, PLGA	vancomycin hydrochloride, ofloxacin, tetracyline hydrochloride	postprint: immersion	INK	drug adsorption and desorption of low temperature 3D-printed ceramic scaffolds	Gbureck et al., (2007) [68]
multi- layered cylinder	PLA	levofloxacin	binder solution	INK	3D-printing of multilayered implant design for bi-modal release profile	Huang et al., (2007) [54]
		levofloxacin, rifampicine				Wu et al., (2009) [128]
multi- layered cylinder	PLA	isoniazid, rifampizin	binder solution	INK	programmed sequentially release of multidrug implant for bone tuberculosis treatment	Wu et al., (2009) [55]
		levofloxacin, tobramycin			for treatment of chronic osteomyelitis	Wu et al., (2016) [56]
scaffold	ceramic, HPMC	vancomycin, heparin, rhBMP-2	ink solution	INK	multijet low-temperature 3D-printing of bioceramic implants with high accuracy of drug deposition to modify the release	Vorndran et al., (2010) [60]
scaffold	MBG, PVA	dexamethasone	preprint: impreg- nation	EXT	3D-printing with new bioactive material MBG for implants with controlled pores, high mechanical strength and sustained drug delivery	Wu et al., (2011) [133]
cylinder	MBG, PHBHHx	isoniazid, rifampicin	preprint: impreg- nation	EXT	3D-printed scaffolds with antitubercular drugs in animal model	Zhu et al., (2015) [129] Li et al., (2015) [130]
scaffold	PCL, PLGA	tobramycin	embedding	heat EXT	3D-printing of scaffold for bone tissue formation and antibacterial properties	Shim et al., (2015) [131]
scaffold	PCL, poloxamine	dexamethasone	HME (syringe/tube)	FDM	dependence of blend ratios on degradation rates and release profiles	Costa et al., (2015) [134]
scaffold	ceramic, PLGA- coating	rifampicin, vancomycin	ink/coating solution, powder mixture	INK	simultaneous local delivery of rifampicin and vancomycin from 3D-printed ceramic scaffolds for bone infection treatment examined in a mouse model	Inzana et al., (2015) [57]
cylinder	CoCrMo	gentamicin	postprint: electro- phoretic deposition	SLM	antibacterial coating of 3D-printed porous CoCrMo bone substitutes	Han et al., (2017) [132]
disc, bead	PLA	gentamicin sulfate	HME	FDM	3D-printing of antibacterial drug doped holloysite nanotubes constructs	Weisman et al., (2017) [44]
scaffold	ceramic, PLGA- coating	rifampicin, sitafloxacin	powder mixture	INK	3D-printing of antibacterial scaffolds for osteomyelitis treatment	Trombetta et al., (2019) [59]
scaffold	PLA	minocycline	postprint: immersion	FDM	3D-printing of scaffold with antibiofilm and osteogenic properties	Martin et al., (2019) [72]
screw, pin, plate	PLA	gentamicin sulfate, methotrexate	HME	FDM	patient-specific fixation implants for localized drug delivery	Tappa et al., (2019) [35]
scaffold	PLA	dexamethasone, prednisolone	pre-/post- print: soaking	FDM	combination of two drug loading mechanisms for different release profiles	Farto-Vaamonde et al., (2019) [135]
disc	PCL	rifampicin	embedding	heat EXT	3D-printing of antibacterial drug containing scaffold at low temperatures	Lee et al., (2020) [137]
cuboid	stainless steel	dexamethasone	postprint: airbrush coating	SLM	coating of 3D-printed stainless-steel implants for slow drug release	Poudel et al., (2020) [63]
screw	PCL, nHA	vancomycin, ceftazidime	embedding	EXT	influence of printing parameter on drug-eluting screws 3D-printed by a solution-technique	Chou et al., (2021) [46]

### 5.4. Antitumoral Devices

Cancer diseases are usually treated with a complex scheme of surgical resection, chemotherapy, radiation or a combination of these methods. Unfortunately, many antitumoral drugs possess poor saturation solubility in aqueous solutions, and an intravenous application is often only possibly after chemical modification or the use of surfactants [138]. Furthermore, systemically applied drugs are typically not targeted to the particular tumoral tissues and may also cause toxic levels in the healthy tissue accompanied by many side effects [138].

Local drug-eluting implants should overcome this and achieve high drug levels in malignant cells. Research on 3D-printing has enabled the manufacturing of implants in several shapes that release chemotherapeutic drugs like 5-fluorouracil, doxorubicin, cytoxan, cisplatin or methotrexate for the treatment of osteosarcoma, pancreatic or breast cancer [47,48,64,69,70]. The biocompatible materials PLA, PLGA and PLC, and also titanium alloy, were used as printing materials for different techniques such as SLM, FDM or other extrusion-based printers [47,48,64,69,70].

Many tests on the biosafety of the printed implants have been successfully performed [48,70] and modifications on the implant design, which are easily and cost-savingly feasible by 3D-printing, were shown to influence the release properties of the incorporated drugs [47,48,69]. Figure 5 shows the findings of Yang et al. [48], who reported that the release of the anticancer drug 5-fluorouracil from 3D-printed PLGA scaffold implants was accelerated with increasing aperture size. The drug release was fast within the first week, followed by slow-release properties which, however, again increased at the end of the experiments due to the self-degradation of PLGA. These and other 3D-printed antitumoral implants proved their efficiency in cell line assays or animal models [47,48,64,70]. Yi et al. and Wang et al. showed higher local than systemic drug concentrations in tested animal models [47,70].

The main facts from selected literature regarding 3D-printed drug-eluting antitumoral devices, including materials used, methods and objectives, are summarized in Table 5.

**Table 5:** List of selected 3D-printed drug-eluting antitumoral devices described in the literature.

Implant Shape	Material	Drug	Drug Loading	Printer Type	Objective	Source
patch	PLGA, PCL	5-fluorouracil	melt mixing	heat EXT	biodegradable patch with high concentrations of anti-cancer drug and modifiable release	Yi et al., (2016) [47]
wafer	titanium	doxorubicin, Apo2L/TRAIL	postprint: droplets	SLM	enhanced bone osseointegration by drug-loaded 3D-printed titanium alloy implants with microrough surface	Maher et al., (2017) [64]
bullet shape	PLA	cytoxan	postprint: immersion	FDM	hollow bullet-shaped implants with modified release properties	Yang et al., (2018) [69]
sphere, cylinder	PLA	cisplatin, ifosfamide, methotrexate, doxorubicin	postprint: immersion	INK	3D-printed multidrug implant for osteosarcoma treatment tested in vitro and in vivo	Wang et al., (2020) [70]
scaffold	PLGA	5-fluorouracil, NVP-BEZ235	embedding	EXT (E-jet)	controlled drug release of 3D-printed implants for orthotopic breast cancer therapy	Yang et al., (2020) [48]

### 5.5. Surgical Meshes

Surgical meshes are often used for the repair of hernias, where an organ leaves its normal position through the holding wall of a cavity. These meshes are usually extruded and knitted to fulfill high demands regarding their long-term mechanical properties, suitable pore size, optimal tissue incorporation, adhesion behavior, good biocompatibility and low risk of infections [139]. Even though there are more than 70 hernia meshes available on the market, the ideal mesh has yet to be developed [139]. 3D-printing could be used as an alternative manufacturing method for further development of optimized surgical meshes.

The on-demand manufacturing of optimal fitting shapes by 3D-printing may make presurgical modifications of commercial surgical meshes redundant. 3D-printed meshes have been developed using different printing techniques and loaded with antibacterial, anti-inflammatory or contrast agents [36,49,51,65,140]. Gentamicin or ciprofloxacin containing meshes inhibited bacterial growth of *E. coli* or *S. aureus* in vitro or were successfully implemented in rat or rabbit models [36,51,65]. Ballard et al. [140] investigated the visibility of 3D-printed meshes by CT imaging. The authors were able to demonstrate the highest CT-visibility for iodine-loaded PLA-meshes compared to those loaded with gadolinium or barium (Figure 6A). Barium-loaded meshes had the only sustained visibility, and signals were not significantly lower after the incorporation of the meshes in agar at a temperature of 37 °C for seven days, mimicking tissue implantation (Figure 6B,C). However, this beneficial imaging characteristic for surgical meshes, as well as for other implants, needs to be inspected after implantation into real tissues to estimate these influences.

A semisolid extrusion 3D-printing technique with a new curing technology was developed by Holländer et al. [49]. The authors printed mesh structures with different pore sizes of medical-grade two-component liquid silicone rubber, which was cross-linked by UV light at 365 nm during and after the printing process. The optimal postprinting curing time was determined to be at least 3 min based on resulting adequate mechanical strength and a high degree of cross-linking of about 95%. The model drug prednisolone was embedded by manual mixing into silicone in concentrations of 0.5–5%, whereas silicone mixtures with drug loadings over 1.5% turned out to be too viscous for 3D-printing without viscosity modifications. Prednisolone was released from the implants over the 28 days of the test period in 4 mL of PBS pH 7.4 with a burst release on the first day. During the inspection of pore sizes of the meshes, it was noticed that the designed quadratic shapes were actually rectangular. A possible reason could be a collapsed bottom layer or widened upper layer in consequence of too low energy of the UV light. Even though this problem may be solved by higher energy variants, this study emphasized the complexity of 3D-printing with challenging or new materials. Nevertheless, the authors report on a promising method for 3D-printing of heat-labile drugs.

The main facts from selected literature regarding 3D-printed drug-eluting surgical meshes, including materials used, methods and objectives, are summarized in Table 6.

**Table 6:** List of selected 3D-printed drug-eluting surgical meshes described in the literature.

Implant Shape	Material	Drug	Drug Loading	Printer Type	Objective	Source
mesh	PLA	gentamicin	HME	FDM	antibacterial surgical meshes from 3D-printer as potential on-demand manufacturing	Ballard et al. (2017), [36]
mesh	silicone	prednisolone	embedding	semi- solid EXT + UV	room-temperature 3D-printing with UV-crosslinking of silicone into different structures and drug loads resulted in different release profiles	Holländer et al. (2018), [49]
mesh	PCL	iodine, gadolinium, barium	embedding	heat EXT	3D-printing of surgical meshes impregnated with contrast agent and characterization of computer tomography properties	Ballard et al. (2018), [140]
mesh	PCL	gentamicin	postprint: droplets	FDM	3D-printed surgical meshes with antibiotics encapsulated in alginate	Calero Castro et al. (2019), [65]
mesh	PP, PVA	ciprofloxacin hydrochloride	preprint: filament soaking	FDM	antibiotic loaded 3D-printed meshes with different pore size, shape and thread thickness of two different materials	Qamar et al. (2019), [51]

### 5.6. Other Devices with Simple Geometry

The shape of a 3D-printed object depends on the predefined computer-created design and is adjustable from simple to complex geometries. Proof-of-concept studies often use simple designs like discs, cylinders or cuboids [37,58,71,74,78,141,142] for the demonstration of the performance of 3D-printing for implants, such as antibacterial efficacy or controllable drug release, by varying implant properties [37,50,58,66,78,141].

The release of antibacterial drugs from 3D-printed implants directly to the infection site, and the prevention of biofilm formation on its surface, could be beneficial for their application in the human body. 3D-printed PLA discs with nitrofurantoin loading from 10% to 30% (*w*/*w*) or PCL scaffolds with a macro and microporous structure loaded with the antibacterial drug cefazolin proved those properties by the growth inhibition of *S. aureus* [37,66].

In 2001, Leong et al. [71] analyzed how the porosity of SLS 3D-printed samples could be controlled by the printing process parameters and determined increased channel width by lower laser power and faster scan speeds. The resulting porosity influenced the release properties of the model drug methylene blue as samples produced with higher laser power released the dye over a longer period.

The release properties of drug-eluting implants are highly dependent on the choice of used material, as release studies of Kempin et al. [141] demonstrated by the use of four different polymers for 3D-printing of hollow cylinders via FDM. All implants released the model drug quinine in 10 mL PBS pH 7.4 over several weeks. However, the drug release of implants with PCL or PLA as the matrix was relatively fast compared to matrices of EC or Eudragit^®^ RS, where only a small amount of the total drug load was released after two or three months. Furthermore, the different drug loads from 2.5% to 15% influenced the released amounts, as well as printability. A higher drug load of 25% quinine resulted in nozzle blockages and limited the possible drug loading of this printing technique for the employed excipients.

Furthermore, the studies of Arany et al. [78] demonstrated the effects of different polymers, sizes and infill ratios on drug release behavior. In this study, the printed dosage forms were cylindrical containers which were used as carriers for the powdered drug, which was manually inserted before the top layers of the carrier were printed. The drug containers were 3D-printed via FDM of PLA, PETG or PMMA in three different sizes, with infill ratios from 0% to 15% and showed different release behavior in dissolution testing in 900 mL phosphate buffer pH 7.4, for example, due to differences regarding available surface areas of different container sizes or hindering infill walls.

Besides the possibility of controlling drug release by adapting the object shape or basic material, additives could be added to the composition for the further adjustment of the sample properties. Distinctive pores were found in 3D-printed bar implants of Salimi et al. [50] after seven days of the release study due to the addition of the water-soluble polymer PEG to the basic printing material of thermo-responsive supramolecular polyurethane.

The presented studies demonstrate current opportunities provided by 3D-printing technology for the manufacture of implants, but the findings which consider printing materials, printing parameters or drug loadings have to be combined in a sensible way for each indication. The knowledge of diseases and application sites is required for implant development regarding the choice of suitable materials and drugs, as well as printing type and parameters to achieve appropriate implant properties including the mechanical behavior, drug release performance and period. 

The main facts from selected literature regarding 3D-printed drug-eluting implants with simple geometry, including materials used, methods and objectives, are summarized in Table 7.

**Table 7:** List of selected 3D-printed drug-eluting implants with simple geometry described in the literature.

Implant Shape	Material	Drug	Drug Loading	Printer Type	Objective	Source
cube	nylon	methylene blue	postprint: immersion	SLS	control of the porosity of 3D-printed construct by varying the printing parameter	Leong et al., (2001) [71]
(hollow) cylinder	PLA	isoniazid	binder solution	INK	drug release from drug-loaded 3D-printed structures	Wu et al., (2014) [58]
disc	PLA, hydroxyl-apatite	nitrofurantoin	HME	FDM	antibacterial feedstock material for 3D-printing	Water et al., (2015) [37]
hollow cylinder	PCL, PLA, EC, Eudragit RS	quinine	HME	FDM	long-term drug release from 3D-printed implants of different polymers and drug loads	Kempin et al., (2017) [141]
scaffold	PCL	cefazolin	postprint: coating	heat EXT	combination of 3D-printing and salt-leaching method for scaffolds with intrastrut microporosity	Visscher et al., (2018) [66]
cuboid	PMMA	flurbiprofen	postprint: super- critical CO_2_	SLA	drug loading of 3D-printed constructs using supercritical carbon dioxide	Ngo et al., (2020) [74]
bar	poly- urethane, PEG	paracetamol	embedding	heat EXT	drug-loaded thermo-responsive supramolecular polyurethane for 3D-printing	Salimi et al., (2020) [50]
disc, bar	PCL	doxycycline, vancomycin, cefazolin	HME	FDM	effect of manufacturing conditions (temperature or UV light) on antibacterial effectiveness	Ranganathan et al., (2020) [142]
	PEG, PEGDA		embedding	SLA simulation		
disc, cylinder	PEGDA	dexamethasone	embedding	DLP	influence of relatively high drug loadings on printability and mechanical properties of DLP 3D-printed constructs	Mau et al., (2020) [52]
cylinder	PLA, PETG, PMMA	diclofenac sodium	powder filling during printing	FDM	in vitro testing of implantable 3D-printed drug carriers	Arany et al., (2020) [78]

### 5.7. Other Devices with Complex Geometry

The first reports of 3D-printing for controlled drug release from implants appeared in 1996 with the studies of Wu et al. [61]. Even back then, the constructed design was complex. The top and bottom layer were printed with PCL as a barrier to the inner PEO matrix. The dyes alizarin yellow and methylene blue, used as model drugs, were manually deposited to their exact position in various arrangements. These early studies demonstrated the high dependence of the drug position, the local composition and accompanying microstructures on release properties, which were controllable by using 3D-printing.

In recent studies Yang et al. [53] developed constructs with internal and external structures via a DLP 3D-printing process that enabled a high level of accuracy. Diverse structures were printed with and without drug loading and demonstrated the influences of the used PEGDA concentrations, plasticizers, photoabsorbers, layer heights and exposure times on the printability and mechanical strength of the resulting objects, whereby the external structure was more influenced by the plasticizer and the internal structures by the photoabsorber. Depending on the surface-volume ratio of the different shapes of the 3D-printed object, diclofenac sodium or ibuprofen were released differently within the 24 h test period, but a burst release within the first hours was observed in all objects. Simple geometries 3D-printed via DLP by Mau et al. [52] demonstrated similar burst release properties, whereas 95% of dexamethasone was released from PEGDA implants within the first 6 h. Furthermore, this study investigated the influence of high drug concentrations even above the saturation solubility of the drug in the photopolymer. Sedimentation was not observed, and the suspended drug was still distributed homogenously in the printed object. However, printability was limited due to the irregular activation of the photoinhibitor in an opaque composition containing 20 g/L dexamethasone resulting in irregularities of shape.

A special extrusion technique of core-shell rods (Figure 7A) was developed by Won et al. [77] for an alternative treatment of retinal vascular diseases instead of monthly injections. A solution of PCL containing bevacizumab for the shell and a poloxamer solution for the core was coextruded using a coaxial nozzle into dual-phasic rods. After rinsing out the poloxamer core, an alginate matrix with dexamethasone was injected as a new core. For intravitreal applications, the simple rod is already a functional shape, but such strands could be shaped into three dimensional devices such as porous scaffolds or spiral stents.

Local drug release rates are controllable by the implant design, which is easily adjustable by the 3D-printing technique. Stewart et al. [75,76] used FDM for 3D-printing of reservoir-type implants, which released the model drugs within a few days or up to several months. The hollow implants were printed completely out of water-soluble PVA or of degradable PLA with windows of PVA in different numbers and sizes (Figure 7B). The model drugs methylene blue, or ibuprofen as a sodium salt or base, were manually filled as powders into the cavity and one implant design was additionally dip-coated with PCL-PEG combinations. The PVA windows dissolved within 25–35 min depending on their surface area, and the drug could exit the implant. Smaller windows seemed to prolong drug release, and the additional barrier of PCL-coating enabled drug release for long-term applications. Further release studies were performed in agarose gels for a better simulation of the physiological conditions compared to the test conditions in 500 mL PBS. The findings of these tests resulted in slower release rates compared to an agitated vessel set-up, presumably due to the fast transport of the dissolved drugs from the media near the implant surface resulting in high drug concentration gradients.

The complex shapes of the presented studies demonstrate the constructional freedom of 3D-printing techniques. For specified application sites, defined suitable structures need to be identified in the future to comply with the corresponding requirements.

The main facts from selected literature regarding 3D-printed drug-eluting implants with complex geometry, including materials used, methods and objectives, are summarized in Table 8.

**Table 8:** List of selected 3D-printed drug-eluting implants with complex geometry described in the literature.

Implant Shape	Material	Drug	Drug Loading	Printer Type	Objective	Source
multi- layered cuboid	PEO, PCL	methylene blue, alizarin yellow	manually deposited	INK	first report of 3D-printing for control of release by modifying drug position, composition and microstructure	Wu et al., (1996) [61]
window implant	PLA, PVA, PEG, PCL	methylene blue, ibuprofen sodium/base	postprint: powder filling	FDM	different implant designs of biodegradable material for controllable sustained drug release	Stewart et al., (2020) [75] Stewart et al., (2020) [76]
multiple shapes	PEGDA, DPPO, PEG	diclofenac sodium, ibuprofen	embedding	DLP	influence of additives, printing parameters and model design on constructs with external and internal structured 3D-printed by DLP	Yang et al., (2020) [53]
rod, scaffold, spiral	PCL, poloxamer, alginate	bevacizumab, dexamethasone	embedding, injected	EXT	coaxial coextrusion for drug-loaded core-shell implant design with gel core	Won et al., (2020) [77]

## 6. Benefits and Challenges of 3D-Printed Drug-Eluting Implants

The presented developments in the field of 3D-printing for drug-eluting implants demonstrate the ongoing progress to more individualized medicine, which is needed for several applications.

Besides open questions on regulatory aspects, the control of the manufacturing process as well as some process-associated characteristics, are still challenging. Necessary postprocessing procedures, such as washing steps or the removal of support structures, could alter the drug load or the optimal fitting of a 3D-printed implant. Furthermore, the sterilization of 3D-printed implants for the avoidance of microbial contamination during implantation is a big challenge because the application of heat or other energetic media by common sterilization procedures, for example autoclaving or the use of ethylene oxide, could influence the product quality. Several components such as thermoplastics would not maintain their shape by thermal treatment, and the influence of reactive gases or steam on the particular components of each composition should be evaluated in future investigations. Guerra et al. studied the effect of different sterilization methods on the properties of FDM 3D-printed stents and stated a strong influence of UV-light on the PCL properties, whereas 70% (*v/v*) ethanol or an antibiotic solution only barely affected the implant material [143]. However, the use of a liquid-based disinfection technology seems questionable in the case of loaded implants, since drug diffusion processes are expected to occur and are probably not controllable. Some 3D-printed implants have been treated with ethylene oxide, low-temperature plasma or UV-irradiation for reduction of microbial contamination prior to in vivo studies in animal models [51,55,66,70]. Nevertheless, further studies regarding the effectiveness of these methods and potential changes of the product are still needed.

For the assessment of the effectiveness of therapies with 3D-printed implants and the knowledge of potential benefits or additional risks compared to conventionally manufactured drug-eluting implants, controlled human trials are needed. However, clinical trials with 3D-printed devices have been commonly performed for anatomical models for preplanning surgeries or guides to aid surgery, but only a very few for therapeutic devices [144]. Positive results regarding the acceptability, safety and effectiveness of 3D-printed products have been shown exemplarily in clinical trials testing oral printlets with customized dosages of isoleucine [145] and during the six-month follow-up of personalized drug-free glass-ceramic implants for the reconstruction of bone defects [146]. Nevertheless, the implementation of predicting in vitro models in combination with biocompatibility assays and further animal testing should be focused on in order to assure the necessary safety of the developed 3D-printed implants and to pave the road for the necessary human clinical trials in the future.

3D-printing is a relatively low-cost method and requires small operation space compared to common manufacturing techniques [14,24,147]. Moreover, the manufacturing of novel geometries in a relatively short time is advantageous for first development steps [14,147]. Even if the throughput of a single 3D-printer is not comparable to that of high-scale manufacturing techniques such as tableting, the manufacturing time for just one individual part can compete with the multiple steps needed in industrial manufacturing. Scale-up by using more printheads or printers simultaneously seems feasible without any process-associated problems and without an investment too high. The most important benefit of 3D-printing of drug-eluting implants is the production of easily adjustable shapes with complex geometries and microstructures. This enables a high level of individualization. The perfect fitting of the implants to the patients´ anatomy is just one aspect [33]. Furthermore, individually needed drug doses depend on various factors, for example, age, weight, gender, genetics or diseases, and by 3D-printing the tailored drug doses can be easily adjusted by modifications of the size, shape, infill percentage of the object or even the feedstock material [13,14,24,33,148]. The administration of multiple drugs in one device could be also achieved by incorporation of different drugs in the starting materials, and different positions of the drugs in the object can offer different release profiles [13,24,33]. The design of controllable complex release profiles for a customized therapy is supported by the wide range of materials for the different 3D-printing techniques and their flexible manufacturing procedure for complex geometries and designs [13,24,33,147,148].

## 7. Regulatory Aspects

A fast-disintegrating porous tablet, namely Spritam made by Aprecia Pharmaceuticals, was the first medicine manufactured by a 3D-printing procedure that was approved by the Food and Drug Administration (FDA) in 2015 and is still the only one that is available on the market [149]. Because of the high complexity of 3D-printed medical products, the small manufacturing quantities and unfeasible standardization if personalization is attempted, the issues surrounding the regulatory challenges have been intensively discussed in the literature in recent years [150,151,152,153,154,155]. The rapidly growing novel technology of 3D-printing of medical devices needs to comply with current regulatory principles, but some of these products do not fit into the current regulatory framework. Therefore, further regulatory adjustments are required. Many drug-eluting 3D-printed implants also face the issue of allocation to the right product category (medical device vs. medicinal product) due to the combinational nature of the products [155]. The same regulatory pathways and manufacturing requirements are applied for 3D-printed and nonadditive manufactured products to guarantee safety for the health and effectiveness of the products [151].

The FDA published a guidance document [150] with their initial thoughts and acknowledged the lack of broadly based experiences and clinical history on this topic. Subjects of their discussion were the materials, the validation of design, printing and postprinting processes, the printing characteristics and parameters, the physical and mechanical assessment of the final product, and biological considerations including cleaning, sterility and biocompatibility. For example, the orientation or location of a printed object during the printing process, or the removal of residual materials or support structures, can affect its properties, and mechanical testing for a unique patient-specific device is impractical as well. The main regulatory challenge for the 3D-printing of medical devices is the validation of the whole process, including the monitoring of printing parameters, testing of the final product properties, and the implementation of acceptance criteria that are suitable for patient-specific devices.

Recently, the company Triastek received an Investigational New Drug (IND) approval from the FDA for the chronotherapeutic drug delivery system T19 manufactured with internal geometric structures to control drug release by a melt extrusion-based 3D-printing process [156]. Years after the approval of Spritam, this underlines the ongoing progress in the development of 3D-printed human drug products under regulatorily accepted conditions. Provided that the clinical trials perform well, T19 may become the second approved 3D-printed medicine, and more may follow in the near future.

## 8. Conclusions

3D-printing of drug-eluting implants is a highly promising manufacturing technique for personalized medicine. The immense diversity of shapes, drugs and materials that have been investigated in the cited studies points out that desired properties can be adjusted for the different needs of several applications and patients. The FDM 3D-printing technique allows for cost and space-effective printing, which offers a large amount of processable materials. This technique enables rapid adjustments for the desired shapes intended for individualized medicine and can be operated easily. Supplemental special requirements, as well as the need for high resolution 3D-printed objects could be implemented by different printing techniques, for example SLS or SLA. Further research needs to be conducted on each formulation and application due to their special requirements, but in the future the perfect shape for an individuals´ physiology, a drug dose for customized needs, suitable mechanical properties and efficient drug release over the intended period could be achievable combined in one implant. Every small step in the development towards this goal will constitute an improvement in the current state of personalized treatments.

## Figures and Tables

**Table 1:** Substantial advantages and disadvantages of different 3D-printing techniques described in the literature.

Printing Technique	Advantages	Disadvantages
INK	+ low costs + fast production + multimaterial printing + no need for supporting structures + low temperature process (suitable for thermolabile drugs) + high porosity	− requires postprocessing (drying, powder removal) − low mechanical properties − requires suitable viscosity of ink − powder wastage
FDM	+ low costs + widely available and compact equipment + multimaterial printing + does not require postprocessing (except for usage of support) + good mechanical properties	− lower resolution − need for supporting structures (depending on printed geometry) − high-temperature process (thermal degradation of drug and excipients) − requires previous filament fabrication
EXT	+ low costs + multimaterial printing + low temperature process (suitable for thermolabile drugs) + high drug loading	− limited resolution (depending on nozzle size) − requires postprocessing (e.g., drying) − low mechanical properties − requires suitable viscosity of semisolids − risk of nozzle clogging
SLA, DLP	+ high resolution and accuracy + fast production	− potential material toxicity − requires postprocessing − need for supporting structures − limited material selection − costly equipment
SLS, SLM	+ high resolution and precision + fast production + no need for supporting structures + highly controllable internal microstructures	− expensive − requires postprocessing − requires suitable particle size − high energy input (degradation of drug and excipients) − wastage of unsintered powder (recycling?)

## Data Availability

No new data were created or analyzed in this study. Data sharing is not applicable to this article.
